# Stakeholders’ perception of factors influencing adoption of a pediatric weight management intervention: a qualitative study

**DOI:** 10.3389/fpubh.2023.1045618

**Published:** 2023-10-13

**Authors:** Desiree Sierra-Velez, Anisha Gundewar, Alicia Persaud, Meg Simione, Ines Castro, Meghan Perkins, Jeanne Lindros, Jeremiah Salmon, Justin D. Smith, Elsie M. Taveras, Lauren Fiechtner

**Affiliations:** ^1^Division of Pediatric Gastroenterology and Nutrition, MassGeneral Hospital for Children, Boston, MA, United States; ^2^Department of Pediatrics, MassGeneral Hospital for Children, Boston, MA, United States; ^3^Division of General Academic Pediatrics, Department of Pediatrics, MassGeneral Hospital for Children, Boston, MA, United States; ^4^Institute for Healthy Childhood Weight, American Academy of Pediatrics, Itasca, IL, United States; ^5^Department of Population Health Sciences, University of Utah School of Medicine, Salt Lake City, UT, United States; ^6^Department of Nutrition, Harvard T.H. Chan School of Public Health, Boston, MA, United States

**Keywords:** pediatric weight management intervention, childhood obesity, implementation science, adaptations, Consolidated Framework for Implementation Research

## Abstract

**Background:**

Childhood obesity is highly prevalent in the United States and disproportionately impacts communities of color and low-income populations; these disparities have worsened during the COVID-19 pandemic. Adoption of effective pediatric weight management interventions (PWMIs) that have been evaluated among low-income diverse populations is needed. The Healthy Weight Clinic PWMI, a package co-developed by the American Academy of Pediatrics and Massachusetts General Hospital, helps health centers establish multidisciplinary Healthy Weight Clinics based on previous randomized controlled trials which demonstrated effectiveness. We sought to identify the factors influencing successful adoption of this PWMI and understand adaptations needed prior to implementation in new sites.

**Methods:**

We interviewed 20 stakeholders, 10 from two health centers in Mississippi where the Healthy Weight Clinic PWMI will be piloted (pre-implementation sites) and 10 from health centers that have previously implemented it (sites in maintenance stages). Separate interview guides informed by the Consolidated Framework for Implementation Research (CFIR) were developed for the pre-implementation sites and those in maintenance stages, including questions related to adaptations of the PWMI in response to the COVID-19 pandemic. Qualitative data analysis was conducted using directed content analysis based on CFIR constructs. Adaptations in response to the pandemic were categorized using Framework for Reporting Adaptations and Modifications-Expanded (FRAME).

**Results:**

In pre-implementation sites, an inner setting facilitator mentioned was a positive learning climate. Characteristics of individuals that can facilitate adoption include staff willingness to learn, valuing evidence-based care for childhood obesity, and culturally and weight-sensitive staff. In terms of patient needs and resources (outer setting), social drivers of health are barriers to adoption, but creative solutions were suggested to mitigate these. Other facilitators related to the intervention included its multidisciplinary model and adaptability. Similar themes were elicited from sites in maintenance stages; adaptations brought on by the pandemic, such as telehealth visits and content modification to align with distancing guidelines and the effects of social isolation were also described.

**Conclusion:**

Understanding the factors influencing adoption of an evidence-based PWMI informs necessary adaptations and implementation strategies required to facilitate nationwide dissemination of PWMIs, with the goal of reaching the populations most at-risk.

## Background

Childhood obesity has a detrimental impact on children’s physical and emotional wellbeing and leads to a higher risk of adverse health outcomes in adulthood (1). The Centers for Disease Control and Prevention estimate that approximately 19% of US children have obesity and an additional 16% of United States children have overweight (2), with worsening rates of BMI increase during the COVID-19 pandemic already documented (3, 4). Children living in low- and middle-income households and those who identify as part of a racial and/or ethnic minority group are disproportionately affected (5, 6), and these disparities have been widening in recent years (7). Moreover, the onset of the COVID-19 pandemic has highlighted these health disparities. Children from low income and minority populations are experiencing greater increases in obesity prevalence (8) and experts anticipated that the school closures, social isolation, and economic devastation caused by the pandemic would only compound on this trend (9). Healthcare delivery and utilization for routine care and non-COVID-19 related diseases have decreased substantially following the onset of the pandemic (10), and greater barriers to identification and treatment of children with overweight and obesity are expected.

Evidence-based pediatric weight management interventions (PWMIs) are necessary to mitigate the impact of obesity on child health, particularly in communities that are at highest risk. Intensive, comprehensive, multidisciplinary interventions are considered to be the most effective treatment for pediatric obesity and are recommended by the United States Preventive Services Task Force (USPSTF) (11, 12). Despite this broad consensus on treatment, participation of children and families in PWMIs remains limited (13), and children at highest risk for obesity have been the least likely to enroll in and complete PWMIs (13, 14). Although the transition to telehealth following the onset of the pandemic has been shown to be feasible (15), delivery of these programs has been affected with suspension of school-based interventions, for example, and experiencing a need to divert resources to pandemic response (16).

The Healthy Weight Clinic (HWC) is a PWMI co-developed by the American Academy of Pediatrics and Massachusetts General Hospital, and funded by the Centers for Disease Control and Prevention, that aims to combine elements of evidence-based interventions tested in Massachusetts that have shown reduction in BMI into a single package. HWC will be piloted at two federally qualified health centers (FQHC) in Mississippi. The findings of this pilot study will then inform the final iteration of the package and the necessary implementation supports for subsequent nationwide dissemination (17, 18). These interventions address childhood obesity at multiple levels through the integration of clinical decision support tools and community-specific behavior change support resources in the primary care setting. In this study, we applied the Consolidated Framework for Implementation Research (CFIR) framework to help identify the key facilitators and barriers to successful adoption of HWC. CFIR is a systematic model for analyzing five constructs that impact effective intervention implementation: intervention characteristics, outer setting, inner setting, characteristics of individuals, and process (19). The use of this framework has been reported across multiple disciplines to understand which adaptations are necessary throughout implementation of an intervention (20). We examined the perspectives of stakeholders from two FQHCs caring for children from families with lower incomes in Mississippi, where HWC will be implemented, as well as stakeholders at sites in Massachusetts where HWC has been successfully implemented and maintained. We also studied the adaptations to HWC resulting from the COVID-19 pandemic.

The engagement of key stakeholders and examination of factors that facilitate and hinder program implementation are essential for successful and sustainable translation of evidence-based community level interventions into new settings (21). Our qualitative examination of identified factors influencing adoption of a PWMI at a range of sites serves to elucidate what may optimize the implementation and adoption of the program at pilot sites, and inform the spread of this program to other locations. The themes identified during pre-implementation stage point to ideas for optimizing implementation and aligning specific needs with utility of the PWMI. Findings that reveal factors of implementation considered during the maintenance stage will have important implications on steering the dissemination and implementation at new sites. Lastly, those modifications that emerged as adaptations to the COVID-19 pandemic may inform continued use and integration of telehealth in PWMIs moving forward.

## Methods

### Participants and settings

To better understand the factors influencing adoption of HWC, we invited stakeholders from pre-implementation sites and sites in maintenance stages to participate in an interview. Participants were considered to be selected from a convenience sample and were contacted via email between November 2019 and May 2021 based on site implementation launch dates. The pre-implementation stage site stakeholders included those from the two Mississippi FQHCs where HWC will be piloted, Aaron E. Henry Community Health Center and Delta Health Center. These pre-implementation interviews were completed between November 2019–January 2020, and November 2019 to December 2019, respectively. The maintenance stage site stakeholders included those from health centers in Massachusetts who had previously worked with two of the interviewers as part of a clinical trial testing one of the PWMIs on which HWC is based on. The maintenance stage site interviews were completed between September 2020 to May 2021.

Participants from pre-implementation sites in Mississippi were introduced to the concept and elements of HWC prior to the interview. HWC is a multidisciplinary PWMI in the primary care setting consisting of a pediatrician, community health worker and dietitian. The HWC also includes support for clinics creating electronic health record-integrated clinical decision support tools, such as BMI alerts and suggested referrals for children with obesity, support and trainings on how to build a community resource guide, educational materials for families including text messages, handouts and videos and staff training resources including asynchronous online modules and a virtual learning collaborative. Patients meet with members of the multidisciplinary team individually and in a group setting over the span of 12 months, receiving >26 contact hours as part of the PWMI, as recommended by the USPSTF. Additional details about HWC have been published (17).

The FQHCs implementing HWC are located in the Delta region of Mississippi. These FQHCs serve a large proportion of children from families with lower income and Black children. Moreover, the state of Mississippi has the second highest prevalence of obesity in children 10–17 years old, and the highest in adolescents and adults, in the United States (22). This is exacerbated in the Delta region where adults and youth report obesity rates exceeding state and national rates (23). The maintenance stage sites are located in Massachusetts where two of the three sites are FQHCs located in communities with a high proportion of children from families with lower incomes, and those who identify as Hispanic. The third site is located within a large urban academic medical center. This pediatric clinic also serves a large proportion of children from similar backgrounds to patients from the FQHCs.

### Interview guides

Two separate interview guides were created, one for pre-implementation sites and one for sites in maintenance stages. The questions generated for the interview guides were adapted from the CFIR interview guide creation tool and themes are shown in Table 1 (24). Additionally, each interview guide included specific questions directed to clinical staff or health center leadership.

**Table 1:** Stakeholders’ perception of the proposed PWMI in the context of CFIR domains and constructs included in interview guide.

CFIR domains	CFIR constructs pre-implementation	CFIR constructs in maintenance stages
Characteristics of individuals	Knowledge and beliefs about the intervention other personal attributes	Knowledge and beliefs about the intervention self-efficacy
Inner setting	Implementation climate Tension for change Compatibility Learning climate Organizational incentives and rewards Relative priority Readiness for implementation Available resources Leadership engagement Access to knowledge and information Networks and communications Culture Structural characteristics	Implementation climate Tension for change Compatibility Readiness for implementation Available resources Leadership engagement Access to knowledge and information Networks and communications Structural characteristics
Outer setting	Patient needs and resources External policy and incentives Cosmopolitanism	Patient needs and resources External policy and incentives Cosmopolitanism
Intervention characteristics	Design quality and packaging Adaptability needs Relative advantage Complexity	Design quality and packaging Adaptability needs Relative advantage Complexity Cost
Process	External change agents Opinion leaders Formally appointed implementation leaders Champions	Engaging Reflecting and evaluating Champions

### Interview procedure

Interviews lasted approximately 45-min and were conducted by five different female members of the research team. Interviewers consisted of two physicians in postgraduate training, one junior faculty physician and two research assistants. All study staff were trained in qualitative methods. Informed consent was obtained at the time of the interview. The appropriate semi-structured interview guide was selected based on each participant’s affiliation with a pre- or post-implementation site. Interviews conducted with participants at the pre-implementation sites were conducted and recorded via telephone. To facilitate remote work during the pandemic, later interviews with stakeholders from sites in maintenance stages were conducted and recorded using the audio-only features of a secure institutional Zoom account. Participants received $75 via electronic check as remuneration. No repeat interviews were conducted and transcripts were not returned to participants for review. All study procedures were approved by the Mass General Brigham Institutional Review Boards.

### Data analysis

The recorded audio files were sent for professional transcription (Landmark Associates). The interview transcripts were coded and analyzed first independently by three of the authors (DSV, AG, LF) using a codebook that was developed utilizing the CFIR guidebook, through a directed content analytical approach (25, 26). Emerging themes were categorized by CFIR domains and constructs as delineated in the interview guides. Additional themes pertaining to other CFIR constructs were included in the analysis as they emerged from the participants’ answers. DSV, AG, and LF met regularly to discuss the findings. During these meetings, and to ensure consistency, variations in coding were discussed and agreements in final coding decisions were reached after reviewing the pertinent CFIR construct definitions. Directed content analysis was conducted until no new codes for each site were identified, indicating thematic saturation was reached. Within each construct, codes were categorized as facilitators or barriers to implementation. Representative quotes for the themes were selected throughout analysis.

## Results

### Participant characteristics

We invited 23 stakeholders, out of which a total of 20 completed the interviews. These include interviews from sites in a pre-implementation stage, and those in maintenance stage. Stakeholders from both the pre-implementation sites and sites in maintenance stages included staff in clinical and administrative roles. Clinical staff included physicians, a nurse practitioner, registered dietitians, behavioral health clinicians, health coaches, and community health workers. Administrative staff consisted of program management, health center leadership, and an electronic health record/information technology specialist. Breakdown of professional roles by site is presented in Table 2.

**Table 2:** Participants’ role in FQHC.

Participants’ role in the health center	Pre-implementation sites	Sites in maintenance stages	Total
Clinical roles			
Physicians	1	1	2
Advanced practice providers	1	1	2
Registered dietitians	1	4	5
Community health workers	-	1	1
Health coach	3	-	3
Behavioral health clinician	1	2	3
Administrative roles			
Program management	-	1	1
Health center leadership	2	-	2
Electronic health record/information technology	1	-	1
	10	10	20

### Findings from pre-implementation stage sites and maintenance stage sites

Barriers and facilitators to adoption of the intervention in the pre-implementation sites and sites in maintenance stages are described below, grouped by CFIR domain and further delineated by stage (pre-implementation or maintenance). The emerging themes are categorized with respect to CFIR constructs. Representative quotes for some of the themes are presented in Table 3.

**Table 3:** Representative quotes from stakeholders by CFIR constructs.

CFIR Domain (Constructs)	Theme	Quotes (site)
Characteristics of Individuals (*Individual state of change, knowledge and beliefs about the intervention*)	Staff is ready for change and has a positive perception of the intervention	*We’re committed to winning the battle of obesity.* (Pre-implementation) *I think this is gonna help because it’s gonna give them that more individualized time with a provider, a dietician, nutritionist, and so forth.* (Pre-implementation)
Inner Setting *(tension for change, compatibility, available resources)*	Program is needed and aligns with institutional values; high importance of evidence-based care for childhood obesity	*I think we’ll definitely be leaders, and really pushing to end some of the obesity here in Mississippi, and not continue to be the bottom of the statistics, and everything* (Pre-implementation) *They’re very proud, and excited and wanted not only—not because of the study, but their patients have an avenue [for] help.* (Maintenance stage)
Outer Setting *(Patient needs and resources, cosmopolitanism)*	Intervention needs to address SDH; connect with local resources in non-stigmatizing, culturally-sensitive manner	*A lot of the fruits and vegetables they are not the freshest. They’re discolored. […] They’re already old before they even get to the supermarket. That is an example of lack of access to fresh fruits and vegetables and whole grains.* (Pre-implementation) *I think we are able to meet the needs of those families who are able to commit and have the motivation, and do not have that additional stressors at home, whether it be resources, financial, food insecurity, mental health, those types of things.* (Maintenance stage)
Intervention Characteristics *(Adaptability, Design Quality and Packaging)*	PWMI needs to adapt to literacy level and culture of community in non-stigmatizing way, multimodal delivery	*It’s just hard for me to come by and then find things that are appropriate for the reading level, education level of what the population that I’m dealing with*. (Pre-implementation) *It’s gonna be tailored to them, and those goals’ll be specific for that child, and you can work on those.* (Pre-implementation)
Process *(Champions)*	Champion role not limited to a single person	*The dietician and the pediatrician [have] the same goal. They are advocating [for] continual maintenance, but it is a permanent […] challenge because we need to fight for what is needed, even though a lot of these transitional administrators do not really get it 100 percent.* (Maintenance stage)

#### Characteristics of individuals

Stakeholders in both sites reported overall positive attributes of their staff members and a commitment to addressing childhood obesity in their communities.

### Pre-implementation stage sites (characteristics of individuals)

Stakeholders reported readiness for change (*individual state of change*) and confidence in the staff’s competence and ability to handle trainings (*self-efficacy*). There was a positive perception of the intervention given its multiple components, and they reported valuing an intervention that leads to timely and tangible results (*knowledge and beliefs about the intervention*). *Other personal attributes* that serve as facilitators of adoption include motivated and positive staff. They also illustrated a high sense of duty for serving as role models to their patients and demonstrating cultural and weight sensitivity (*other personal attributes*).

### Maintenance stage sites (characteristics of individuals)

Stakeholders reported that there was an importance placed on collaborative care, motivation to work with this population, and that staff members valued the ongoing learning *(other personal attributes).* Facilitators in this intervention were prior experience and knowledge of behavior change state, and confidence in continuing intervention (*self-efficacy*). Knowing that the evidence gathered from this innovation is a way to improve future iterations served as a motivator *(knowledge & beliefs about the intervention)*.

### Inner setting

Overall, the culture and desire to change clinical practices to address childhood obesity more effectively are important facilitators for implementation of HWC. Stakeholders also identified available resources and those that would be needed to ensure a successful implementation of HWC.

### Pre-implementation stage sites (inner setting)

In line with the characteristics mentioned above, the staff members believe that credibility and role modeling are essential for the FQHC to create a culture of healthy living that resonates with the community (*culture*). In turn, gaining recognition as a key instrument in improving the community’s health is valuable to the FQHC beyond monetary rewards (*organizational incentives and rewards*). They also noted that they would like this to be the case in all their locations and school-based clinics (*structural characteristics*).

Within the implementation climate, there was importance placed on providing evidence-based care for children with obesity. They expressed the need to change current practices to align with evidence, and to overhaul the existing referral system (*tension for change*). Stakeholders reported that clinical decision support tools, such as a BMI alert, along with recommended orders for further diagnostics and referrals, would improve their workflow (*compatibility*). Stakeholders also reported previous experience with referrals to healthy lifestyle interventions and text messaging campaigns; however, there are difficulties keeping up to date phone number records which is a potential barrier (*compatibility*). To better support the implementation of HWC, stakeholders aim to foster an environment that is conducive to learning (*learning climate*) and communicate clear goals (*goals and feedback*).

One key aspect of the FQHC’s readiness for implementation is their sense of counting on their leadership (*leadership engagement*). One of the centers reported that the accessibility of a large fitness center and a fitness trainer becoming available before the program starts serve as facilitators for adoption (*available resources*). Additionally, access to personnel such as Women Infants and Children (WIC) staff, community health workers (CHW), and Diabetes Prevention Program-trained staff that can help implement the program is another facilitator (*available resources*). However, potential barriers to adoption include high staff turnover and expected efforts by staff to ensure participants follow-up with program (*available resources*). Participants believe that offering continuous asynchronous training opportunities is key to continuing the program. In order to make implementation smoother, stakeholders reported having clear workflow expectation and print materials and guidelines to refer to is essential (*access to knowledge and information*).

Stakeholders also recognize several elements and strategies to further support implementation: utilization of current CHWs as referral sources, informal meetings for planning, clinician meetings to promote use of the intervention, and using existing quality improvement initiatives (*networks and communications*). They also highlighted the importance of ensuring relevant information is documented so that the staff can be aware of and address barriers that patients may be facing (*networks and communications*).

### Maintenance stage sites (inner setting)

Staff members were motivated and positive about implementing this intervention (*culture*). They found that pediatric providers in the community had a desire for this type of intervention (*implementation climate*). With regards to scheduling, stakeholders reported minimal issues with selecting a specific day and time, because HWC was scheduled only once a week. Stakeholders reported that implementing this program was a smooth transition from a previous program and aligned with the values that were already in place (*compatibility*).

Stakeholders reported that team dynamics adjusted. There were shifts in leadership roles based on need. The teams noted that there was a sense of autonomy with regards to time allocation and learning about tools to enhance the program (*learning climate*). The constant communication between staff members was a key facilitator to achieving the goal of running a HWC (*networks and communications*). There was a clear commitment of leadership to meet the program and patients’ needs (*leadership engagement*). Stakeholders reported that a program like this was needed at the time but especially after the impact of COVID-19 on health-related behaviors (*tension for change*).

It was noted that additional training regarding children with Avoidant/Restrictive Food Intake Disorder and autism outside of the HWC would have been helpful as it requires a very different approach for goal setting and making health modifications. Stakeholders expressed that communication with clinics that launched successful programs could have been an asset (*access to knowledge and information*).

The stakeholders reported that they had the physical space, staff, materials, and opportunities for this intervention to be successful; however, keeping pediatric providers on staff was difficult. There were not enough and it was challenging to hire people (*available resources*). The complexity of the administrative structure delayed changes being made (*structural characteristics*).

### Outer setting

Stakeholders in both sites shared an understanding of the importance of ensuring participants and their families access the community resources they need, such as healthy food and behavioral health support, for the success of HWC in their communities.

### Pre-implementation stage sites (outer setting)

The major themes regarding the outer setting revolve around *patient needs and resources.* Stakeholders identified a host of barriers that need to be mitigated to ensure the success of implementing HWC. These barriers include lack of access to affordable healthy foods, limited transportation, lack of physical activity gear and lack of safe spaces and opportunities for being active, which in turn result in excessive screen time. Additionally, stakeholders believe that buy-in from the community could be difficult. Obesity is so prevalent that many patient families do not worry about it until obesity-related complications develop. Moreover, the food culture does include the consumption of fried food and fast foods. Stakeholders suggested some strategies to mitigate these barriers. For instance, they recommend marketing, texting families, monitoring help with home visits, and incentivizing participation to encourage families to join the intervention. They also suggested linking families to the appropriate resources to address socioeconomic drivers of health. Participants believed this would be important because at the time of these interviews, WIC benefits in Mississippi were required to be claimed at specific centralized locations rather than community grocery stores (*external policy and incentives*).

To further support these needs, stakeholders suggested that community partnerships could be leveraged creatively when resources are limited *(cosmopolitanism)*. Another suggestion was to engage farmers in the Mississippi Delta Region to serve as a source of fresh local produce for families that need it. Lastly, there is a desire for the FQHC to become recognized as a key player in improving families’ health *(peer pressure).*

### Maintenance stage sites (outer setting)

Staff members reported that connectedness to other organizations supported implementation, and spread knowledge outside of the medical encounter (*cosmopolitanism*). Stakeholders suggested that those looking to implement this intervention should seek engagement from outside foundations for support of non-reimbursable services and work toward maximizing billable services (*external policy and incentives*).

Previous experience working with diverse populations was an important facilitator. There was a need for behavioral health support for co-existing depression, anxiety and disordered eating. Staff members reported that once participants started opening up, they shared intimate aspects of their life they did not even realize they were ready to share. A close connection to local food banks and resources that support safe outdoor physical activities helps families receive or access necessary resources after referral. It is important to be cognizant that transportation may be a barrier to follow up and that session times and lengths may need to be adjusted to better serve patient needs (*patient needs and resources*).

### Intervention characteristics

The design quality and packaging of HWC was one of the most important qualities mentioned by stakeholders. However, they stressed the importance of ensuring the content adapts well to the context of their respective communities.

### Pre-implementation stage sites (intervention characteristics)

As part of the interview, stakeholders were introduced to elements of the HWC package. Stakeholders reported an overall positive perception of the program (*intervention source*) which can help facilitate adoption. The participants perceive the multiple components of the package and the focus on multidisciplinary individualized care as a facilitator to implementation (*design quality and packaging*). Stakeholders appreciate that the package supports the development of lifelong healthy behaviors, with clear and concrete examples, and that text messaging is utilized for positive reinforcement (*design quality and packaging*). Other aspects of the package that serve as facilitators include the availability of repeated and consistent trainings, community resource guides, and resources addressing bullying and adverse childhood experiences (*design quality and packaging*). When compared to other interventions, participants believe that HWC is advantageous due to its interactive curriculum, family centeredness, and ability to be sustained with available resources (*relative advantage*).

Participants mentioned that adapting the content to the literacy level and culture of the participants is important (*adaptability*). They would also advocate for multimodal delivery of the content as some families may not be able to commit to the full program; they suggested having make-up sessions and use of video, telephone and text messaging to deliver content (*adaptability*). Additionally, they reported that having a CHW as part of the team is helpful for adapting and tailoring the package (*adaptability*).

### Maintenance stage sites (intervention characteristics)

Stakeholders reported that staff members were able to train nurses to work on goal setting with patients when the other medical providers were not available. It was noted that adaptations were made to materials to make them less stigmatizing around nutrition choices. There were telehealth adaptations made to accommodate the needs created by COVID-19 (*adaptability*). Telehealth added layers of complexity given the multidisciplinary model (*complexity*). Moreover, there is a need to identify avenues for sustainability when it comes to cost; self-funded interventions are more likely to be supported by leadership (*cost*).

Stakeholders reported that the materials were high quality; however, there is a need to include site-specific adaptations. Incentives for participation such as healthy snacks and jump ropes accelerated engagement, and group visits were highly valued. Not being able to conduct in-person group visits due to COVID-19 was a major threat to successful implementation (*design quality and packaging*). The inclusion of behavioral and emotional wellness aspects was a huge advantage to this intervention in comparison to other PWMIs (*relative advantage*). This package allowed for trials of smaller-scale changes. When stakeholders began implementation they were able to see what worked and respond to what did not work (*trialability*).

### Process

Implementation champions are considered as extremely valuable to the promotion and success of the intervention. For sites in maintenance stages, frequent meetings and tracking of outcomes were beneficial throughout implementation.

### Pre-implementation stage sites (process)

Stakeholders reported confidence in a clinician champion’s ability to promote the intervention (*champions*). They also recognized that there is a need to receive buy-in from specific people in leadership roles (*opinion leaders*).

### Maintenance stage sites (process)

The stakeholders noted that having prior experience with managing aspects of childhood obesity was helpful when recruiting staff (*engaging*). Having a champion was key (*champions*). Meeting in advance with team members and conducting periodic logistic meetings served useful throughout the intervention (*planning*). The COVID-19 pandemic forced sessions to meet less frequently (*executing*). Staff members tracked behavioral change outcomes by reviewing previous goals and seeing where patients were with regards to those goals (*tracking behavioral change outcomes*). They were able to track clinical outcomes by assessing BMI and lipid profiles (*tracking clinical outcomes*). They tracked retention and engagement by keeping attendance and tracking no-show rates for one-on-one visits (*tracking retention/engagement*).

### Adaptations at maintenance stage sites in response to the COVID-19 pandemic

Upon the declaration of a state of emergency in Massachusetts in March of 2020, healthcare delivery for non-emergent care had to adapt to the circumstances by shifting to telehealth. Health centers offering PWMIs adapted similarly. We were able to identify adaptations that were brought on by the COVID-19 pandemic. We categorize them by using Framework for Reporting Adaptations and Modifications-Expanded (FRAME) (27), an implementation science framework to categorize modifications to evidence-based interventions. These are presented in Table 4.

**Table 4:** Modifications to PWMI following onset of the COVID-19 pandemic, classified using FRAME.

Modification	Decision-makers	Modification category and level of delivery	Nature of modification and fidelity	Reasoning	Examples
Telehealth delivery of intervention	Political leaders, administration, intervention team	Contextual; organizational, clinic and individual levels	Changes in packaging; fidelity consistent	Existing mandates, historical context, access to resources of participants, literacy and education level of participants, emergent circumstances	State Department of Public Health’s mandate to provide virtual care, participant’s availability of internet access for telehealth, technological literacy of participants, flexibility on type of delivery given individual circumstances
Virtual group visits	Administration, intervention team	Contextual; clinic and provider-levels	Changes in packaging; fidelity consistent	Existing mandates, historical context, available resources, clinical judgment, perception of the intervention	Group visits offered via videoconferencing platform due to perception of these as key to the intervention
Sharing of educational materials	Intervention team, individuals	Contextual; clinic, provider, and individual levels	Changes in packaging, tailoring, condensing; fidelity consistent	Existing mandates, historical context, access to resources of participants, literacy and education level of participants, emergent circumstances	Use of screen sharing to show materials, emailing PDF handouts, texting shorter versions of educational materials, use of patient portal, postal mail
Handoff	Intervention team	Contextual; clinic level	Substituting; fidelity consistent	Existing mandates, historical context, available technology	Using electronic means of communication instead of in-person communication to facilitate handoff to promote physical distancing
Curriculum	Intervention team	Content; clinic-level	Adding elements, removing elements, substituting, tailoring; fidelity-consistent	Historical context, clinical judgment, perception of the intervention, access to resources, crisis or emergent situations, motivation	Removed recommendations that did not align with physical distancing guidelines such as encouraging indoor physical activity instead of group sports, substituted bullying content for general emotional well-being and stress management curriculum, acknowledged increase in screen time due to remote learning and ways to counteract
Healthy Weight Clinic structure	Administration, intervention team	Contextual; clinic-level	Loosening structure, removing elements; fidelity-inconsistent	Historical context, available resources, competing demands, time constraints	Pausing group visit offerings in some settings, physician-only visits, asynchronous visits with behavioral health clinician or dietitian

All adaptations due to the COVID-19 pandemic occurred during the maintenance phase. Thus, they were considered reactive, with the goals of increasing reach and engagement of the intervention. Most adaptations were decided by the intervention team; however, changes to the structure of the PWMI were also influenced by health center administration and public health mandates. The majority of the modifications were contextual, at multiple levels of delivery, and primarily consisted of fidelity-consistent changes in the packaging of the intervention. The intervention’s content had to be modified as well to align with public health experts’ recommendations for reducing the spread of COVID-19. The changes in content included eliminating, adding and substituting content as appropriate while remaining fidelity-consistent. Notably, changes in the HWC structure by eliminating the synchronous multidisciplinary approach, which were done in response to staffing constraints, would be considered fidelity-inconsistent.

Access to resources by participants, their literacy level, and emergent circumstances also influenced the change to telehealth delivery and the methods of sharing educational materials with participants. Access to health center resources and technology also impacted the handoff communication methods and the change in the structure of the HWC. During this period of uncertainty, perception of the intervention and clinical judgment guided decisions regarding virtual group visits.

## Discussion

In this study we were able to recognize common multi-level factors influencing adoption of a PWMI across sites in different stages of implementation. We identified that counting on highly motivated individuals with confidence in their abilities to implement HWC in their community is a key facilitator to implementation and aligns with the individuals’ sense of duty to their community and desire to make a positive impact in children’s health. This is compatible with an institutional commitment to change clinical practice to address childhood obesity in a more effective manner, demonstrated by the ease with which stakeholders identified existing and needed resources for successful implementation of HWC. Specifically, stakeholders understand their communities and the need for distinct resources such as better access to affordable healthy food options and behavioral health support. Stakeholders recognize that leadership support and identifiable champions are essential to implementation success. Lastly, stakeholders appreciate the quality and packaging of HWC but recognize that HWC needs to be able to adapt well to their communities for lasting change.

Our findings suggest that program adaptations should ideally be incorporated as early as during the planning stages of implementation. Specific adaptations brought on by the COVID-19 pandemic can also serve to highlight the role of telehealth in PWMIs moving forward. Our findings of common multi-level factors influencing adoption of a PWMI between both sites are encouraging given the success of the PWMI in improving BMI and health behaviors at the sites in maintenance stages (28).

Existing literature supports the use of systematic approaches to implementation, such as using the Consolidated Framework for Implementation Research (CFIR), to describe the factors that influence implementation of evidence-based practices and produce generalizable knowledge about implementation science methods. By using this framework, we were able to understand the needs of the new sites launching HWC and react accordingly. We anticipate that following this approach will help with launching HWC in new sites as we move into further dissemination stages. Furthermore, by categorizing modifications made to the PWMI at sites in maintenance stages using FRAME we can gain a better appreciation of the circumstances driving program modifications. This can help us understand the downstream effects of these modifications on implementation and clinical outcomes. Stakeholders in both sites concurred that there is a pressing need for PWMIs in their communities. They believed that HWC would help address this need, which is likely to grow with reported worsening rates of childhood obesity following the onset of the COVID-19 pandemic (3). Reducing these needs has been shown to correlate with high levels of acceptance of new programs in the local healthcare community (29). This tension for change serves as a facilitator to implementation.

The attention of the PWMI to patient needs and resources (CFIR Outer Setting) was one major aspect brought up by stakeholders in both groups. This reflects the heightened awareness of the medical community to the social drivers of health (SDH) and how they influence health disparities, particularly following the COVID-19 pandemic. The AAP has called for addressing SDH given its deleterious effects on childhood health (30–32); however, gaps between recognition of SDH and intervention continue to exist. Clinical-community partnerships have been proposed as one strategy to address SDH needs (33). In the realm of childhood obesity prevention and interventions such partnerships have been reported, particularly in the school setting (34). One example is the modification of school meals resulting in improved dietary behaviors (35). It is encouraging that pre-implementation sites’ stakeholders recognized potential local partnerships that, if leveraged, could lead to increased recruitment and engagement, while contributing to sustainability of the PWMI beyond the pilot phase.

To contextualize HWC to each community, placing a greater emphasis on the role of the CHW as part of the team would be important. This role could serve a dual purpose of helping address SDH while ensuring HWC delivers culturally relevant care, another aspect that stakeholders appreciate as crucial during implementation. Despite limited data, integration of CHWs into healthcare teams has been suggested to contribute to improving clinical outcomes (36). By collaborating with stakeholders throughout the implementation process, we maximize our understanding of the nuances of the community and brainstorm necessary adaptations while ensuring fidelity to the evidence-based intervention. Adaptations of evidence-based interventions are commonly reported (37), particularly those that aim to adapt the intervention to a new setting and make it more culturally-relevant to the target community.

Another key facilitator that was identified is that staff members in these health centers are motivated, creating a greater degree of self-efficacy in the capability of the center to initiate and continue the intervention. Self-efficacy and a positive perception of the intervention have been linked to positive implementation outcomes; notably, program supports can impact this self-efficacy (38). Individuals with higher self-efficacy may also have less training needs over time (39). Champions were quintessential to the success of the intervention, in keeping with the implementation science literature (40).

There are barriers specific to the pre-implementation sites that need to be addressed. One cultural aspect that was mentioned by stakeholders is that many people in this geographical region do not worry about obesity until complications develop since obesity is very prevalent within the community. Studies have shown that many parents tend to underestimate their child’s weight status (41, 42) and this misperception is more common in populations with lower incomes and Hispanic and Black populations (43). This could impact the acceptability of HWC in the community and requires that clinicians be trained to effectively educate families about childhood obesity.

At the policy level, another concern brought up by stakeholders from the Mississippi pre-implementation sites was that at the time of the interviews, the state’s WIC policy did not allow for benefits to be claimed at regular grocery stores. The prevalence of household food insecurity in Mississippi is 15.3% from 2018 to 2020, or around 1.2 million households, which is the highest in the country (44). Given the known links between food insecurity and obesity (45), recent modification of the state’s WIC program which now allows cash benefits to be redeemed in retail stores may help alleviate food insecurity. The expansion of other federal programs would also be of benefit to decrease food insecurity.

An additional barrier identified by stakeholders pertains to the sustainability of this intervention. High rates of staff turnover could impair the ability of HWC to be sustained. Sites in maintenance stages were able to address this by promoting flexibility in staff roles to help bridge the gaps created by turnover. In pre-implementation sites, stakeholders recognized continuous training opportunities as a target for ensuring the sustainability of the program. In the nursing literature, achieving a higher sense of accomplishment, having interest in the job and accessing opportunities for development were identified as essential for occupational satisfaction (46). While staff at the pre-implementation sites are motivated, leaders should leverage tools to maintain or enhance this motivation as the program launches and continues, particularly as the healthcare system experiences high turnover in the wake of the pandemic.

A potentially positive effect of the COVID-19 pandemic is that it propelled adaptations to the curriculum and the incorporation of telehealth options as part of PWMIs. Although randomized controlled trials are needed, previous studies have reported the feasibility of conducting weight management interventions via telehealth and decreasing no-show rates (15, 47). Yang et al. comment on the use of telehealth as a tool to manage patients with type 2 diabetes during the COVID-19 pandemic. While the authors recognize benefits such as improved glycemic control and cost-effectiveness, they also state limitations such as many clinicians lacking experience delivering telehealth, technical difficulties, reliance on equipment that may fail in low power situations, and more importantly, the inability to conduct key portions of a physical exam that could help identify serious complications from diabetes. In the context of HWC, where education comprises a major part of the intervention, a hybrid model with regular in person examinations by a clinician alternating with educational sessions and visits with a dietitian via telehealth may be appropriate (48). A recent systematic review suggests that these hybrid models may be ideal, providing flexibility by offering virtual follow-up visits after an initial in-person evaluation (49). Individuals with known obesity-related complications may require more frequent in person visits than others for monitoring of these complications. Nevertheless, including telehealth offerings in the package can help balance the transportation barrier that was reported by the pre-implementation sites stakeholders. However, it is important that the sites and leaders understand the technological literacy of the participants as well as the ease of access to the internet and availability of necessary equipment to be able to successfully use virtual platforms.

One of the major strengths of our study is that we engaged stakeholders at the FQHCs before implementation commenced to better understand local needs and resources. These stakeholders have a variety of roles in the health centers, which helps identify different perspectives. Multiple studies have previously supported the use of implementation science methods prior to implementation to maximize success of the interventions. By utilizing the CFIR domains and constructs, we were able to categorize these factors influencing adoption systematically and compare them to the experiences at the sites in maintenance stages. We additionally incorporated implementation strategies and targeted outcomes into the Implementation Research Logic Model (50) to further plan for and guide our efforts to implement the HWC in the Mississippi FQHCs. This created a complete conceptual model of which strategies would be used to overcome barriers identified by stakeholders (17). Moreover, including adaptations suggested by pre-implementation site stakeholders could help create a sense of ownership of the intervention among staff in the Mississippi FQHC’s and keep them engaged to continue to offer HWC to their communities past the study period. As HWC moves into dissemination stages, understanding these influencing factors as categorized using CFIR, and with associated evidence-based strategies to overcome barriers and leverage facilitators, can help facilitate adoption in new sites.

This study, in part due to its qualitative design, does have limitations. First, despite reaching thematic saturation with the number of interviews conducted, the smaller sample size may not fully reflect the experiences of the entire team or of those that may join later. The smaller sample size reflect the staff turnover at the maintenance stage sites and the fact that the pre-implementation sites were in the process of recruiting staff during the study period. This precludes randomization of the sample to minimize bias. Notably, the interviews of stakeholders at maintenance stage sites occurred after the onset of the COVID-19 pandemic, with adaptations at these sites already underway. These circumstances could have potentially influenced stakeholders’ recollection of initial implementation and their perception on how that process occurred when compared to the significant obstacles to delivery of the PWMI brought on by the pandemic. We also do not have data on the perceptions of stakeholders at the pre-implementation sites following the onset of the pandemic. It is possible that the needs of the community changed substantially since the interviews were conducted, especially given the disproportionate morbidity and mortality Black individuals experienced as well as the economic impact on already under-resourced communities. Additional sets of interviews could be conducted to understand changes that may need to occur to promote successful transitions in hiring and training of new staff, and supporting existing staff, as these factors may relate to clinic implementation. In our study, we did not include the perspectives of potential participants in the HWC pilot study, which could have helped refine the adaptations that stakeholders at the sites have already identified or include new ones. Another potential limitation is our use of different interview guides based on whether the sites had implemented a PWMI or not as this risks provision of more heterogeneous data. However, our decision to use CFIR to create interview guides and code interview transcripts serves our aim to reduce the risk of bias. Moreover, we ensured different researchers coded transcripts individually before meeting as a group to avoid bias. Lastly, although the communities served by the FQHCs in Massachusetts and Mississippi are both considered low-income, the racial and ethnic differences between these communities may suggest that different approaches to mitigating barriers to adoption, participant engagement and retention may be necessary. In particular, programs need to consider the deleterious effect that racism and negative experiences with healthcare has had on promoting medical mistrust within the Black community, which has been linked to adverse effects on physical and mental health indicators (51), and delays in seeking medical care (52).

By using systematic implementation science methods, we anticipate maximizing the uptake of this intervention and identifying areas that will help disseminate HWC nationwide. This way, we can help children with obesity and their families access the services and tools they need, and in turn decrease the prevalence of obesity and its complications in the populations that are at most risk.

## Conclusion

Understanding the barriers and facilitators to the adoption of an evidence-based PWMI from the pre-implementation through the maintenance stages can help researchers incorporate adaptations to these programs, while supporting the implementation process in new sites with the goal of successful uptake, sustained delivery, and eventual scale up and spread. The design quality and packaging and adaptability of the intervention, as well as the attention of the intervention to patient needs and resources were the most important constructs for stakeholders to consider throughout the process of implementation. Dissemination of further iterations of HWC and other evidence-based interventions benefit from utilizing CFIR to understand factors influencing implementation and adapt the intervention as needed.

## Contributions to the literature

- Uptake of evidence-based pediatric weight management interventions (PWMIs) has been limited, particularly in communities with lower income and minority populations.
- Few studies have described the factors influencing adoption of PWMIs in new settings, while simultaneously evaluating sites in maintenance phases to facilitate implementation in these new settings.
- Understanding these factors is key to adapting interventions in real-time during the implementation process in order to ensure PWMIs reach those children at highest risk of obesity and related comorbidities.

## Data availability statement

The raw data supporting the conclusions of this article will be made available by the authors, without undue reservation.

## Author contributions

DSV and LF developed the study design and the interview guides. DSV, LF, and AG conducted data collection and analysis. DSV, AG, and AP conducted the literature search. DSV, AG, AP, and LF drafted the manuscript. MS, IC, MP, JL, JS, JDS, and ET contributed to data interpretation and manuscript review and editing. All authors contributed to the article and approved the submitted version.
